# A Straightforward Access to New Amides of the Melanin Precursor 5,6-Dihydroxyindole-2-carboxylic Acid and Characterization of the Properties of the Pigments Thereof

**DOI:** 10.3390/molecules27154816

**Published:** 2022-07-27

**Authors:** Rita Argenziano, Marina Della Greca, Lucia Panzella, Alessandra Napolitano

**Affiliations:** Department of Chemical Sciences, University of Naples “Federico II”, Via Cintia 21, I-80126 Naples, Italy; rita.argenziano@unina.it (R.A.); dellagre@unina.it (M.D.G.); panzella@unina.it (L.P.)

**Keywords:** catechol, DHICA amides, melanins, pigments, oxidative polymerization, chromophores, dermocosmetic formulations

## Abstract

We report herein an optimized procedure for preparation of carboxamides of 5,6-dihydroxyindole-2-carboxylic acid (DHICA), the main biosynthetic precursor of the skin photoprotective agents melanins, to get access to pigments with more favorable solubility properties with respect to the natural ones. The developed procedure was based on the use of a coupling agent (HATU/DIPEA) and required protection of the catechol function by easily removable acetyl groups. The *O*-acetylated compounds could be safely stored and taken to the reactive *o*-diphenol form just before use. Satisfactorily high yields (>85%) were obtained for all amides. The oxidative polymerization of the synthesized amides carried out in air in aqueous buffer at pH 9 afforded melanin-like pigmented materials that showed chromophores resembling those of DHICA-derived pigments, with a good covering of the UVA and the visible region, and additionally exhibited a good solubility in alcoholic solvents, a feature of great interest for the exploitation of these materials as ingredients of dermocosmetic formulations.

## 1. Introduction

In recent years, catechol systems including those occurring in natural sources have attracted much interest for their versatile chemistry and, particularly, the redox reactivity [1,2]. The oxidative conversion of these compounds is a process brought about by phenolases in nature leading to colored polymeric pigments [3,4,5], but a variety of other oxidants and even aerobic slightly alkaline conditions have been largely used for preparation of bioinspired pigmented materials including notably polydopamine [6,7,8] for application in the fields of organoelectronics [9], sensors [10], tissue regeneration [11,12], and bioadhesives [13]. Of particular interest in this connection are melanins [14,15], the polymeric pigments responsible for the dark pigmentation of hair, skin, and eyes of humans and non-human mammals, that are generated from tyrosinase-catalyzed oxidation of tyrosine [16]. The final pigments are generally regarded as highly phototoprotective thanks to their broad chromophore spanning over the whole visible region, but a variety of other properties, e.g., photothermal response [17] and antioxidant activity [18], have been documented [19,20]. Studies carried out on model pigments prepared by oxidation of the ultimate monomeric precursors, namely 5,6-dihydroxyindole (DHI) and 5,6-dihydroxyindole-2-carboxylic acid (DHICA), have shown that DHI melanins are black in color on account of an intense absorption over the visible region, while DHICA melanins are lighter in color, exhibiting an intense absorption in the UVB/UVA range, the most dangerous portion of the solar spectrum, and a moderate absorption in the visible region, together with a marked antioxidant activity [21,22]. These features would point to the exploitation of DHICA pigments as active ingredients in dermocosmetic formulations with a combined sunscreen action and protection toward cellular damages induced by UV light and oxidative stress. Recently, we investigated the possibility of tuning the properties of DHICA melanins for dermocosmetic purposes through modification at the carboxylic group. Though exhibiting more favorable solubility properties with a good antioxidant profile, melanins from DHICA methyl ester failed to provide an intense covering of the visible region, an observation that coupled with consideration of the poor metabolic stability of the ester functionality led us to consider alternative derivatives [23,24].

Amides in particular appeared as good candidates because they are stable under physiological conditions, and the electron donor character of the carboxamide group is expected to favorably affect the chromophore of the final melanin pigment with respect to carboxymethyl group. Moreover, proper choice of the amino component should warrant a good solubility in organic solvents of the final melanin-like pigments, which is a critical requisite for their full exploitation in dermocosmetic formulations. 

Amides of DHICA have been previously prepared and described as inhibitors of HIV’s integrase [25,26]. Yet, amidation of a carboxylic acid featuring additionally an *o*-diphenol functionality poses several difficulties, since the OH groups may act as competitive nucleophiles, but mostly because the reaction conditions are typically alkaline, favoring oxidation of the catechol, so that rigorous air exclusion is required. Recently, a successful synthetic protocol applied to caffeic acid reported the protection of the OH groups before the amidation step [27]. Other studies on different catechol systems described a variety of reaction conditions using different coupling agents (e.g., N,N′-dicyclohexylcarbodiimide (DCC)/4-dimethylaminopyridine (DMAP) [28], or ethyl-3-(3-dimethylaminopropyl)carbodiimide (EDC)/hydroxybenzotriazole (HOBt) [29] or benzotriazol-(1-yloxy)tris (dimethylamino) phosphonium hexafluorophosphate [30,31]). However, all these procedures afforded the target amides in low to moderate yields also because of the troublesome purification steps needed. 

We report herein a straightforward facile synthetic route that allowed access to amides and diamides of DHICA in very good yields. The scope of the reaction was explored by use of aliphatic and aromatic amines, as well as of 1,n-diamines. These latter are also of particular interest to form pigments with peculiar supramolecular arrangements arising by coupling of the indole moieties that are linked together by flexible chains of different lengths. The chromophoric and solubility properties of the pigments formed by oxidation of the different amides synthesized were investigated in comparison with those of the pigment from DHICA.

## 2. Results

### 2.1. Synthesis of DHICA Carboxamides 

The melanin precursor DHICA representing the starting material of the synthetic procedure was obtained on gram scale by a straightforward one pot procedure involving ferricyanide oxidation of 3,4-dihydroxyphenylalanine (DOPA) as previously described [32].

In our initial experiments the synthesis of DHICA butanamide was attempted using EDC, HOBt, and triethylamine (TEA) in dry DMF, at room temperature for 18 h under controlled atmosphere according to a protocol reported in literature to prepare caffeic acid amides that required prior acetylation of the catechol functionality [33]. 5,6-Diacetoxyindole-2-carboxylic acid (DAICA) was therefore prepared by acetic anhydride/pyridine (Py) treatment overnight followed by 1:1 *v*/*v* methanol/water under reflux to hydrolyse the mixed anhydride formed at the carboxyl group. HPLC analysis of the reaction mixture after amidation revealed partial conversion of the starting product to another component that did not show the molecular ion peak expected for the amide on LC/MS analysis. This latter analysis also showed substantial formation of the *N*-acetyl butylamine likely arising from displacement of the acetyl group from the catechol function of DAICA by the amino group.

**Figure 1:**
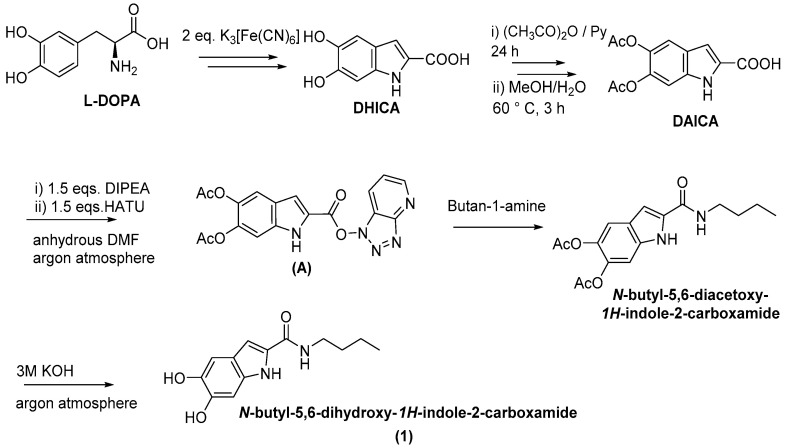
Synthesis procedure for DHICA amides. Shown is also preparation of DHICA from DOPA.

Hence, different synthetic strategies were explored. After several attempts, the best results were obtained pursuing a protocol reported in the literature for solid state peptide synthesis based on the use of 1-[bis(dimethylamino)methylene]-1*H*-1,2,3-triazolo[4,5-b]pyridinium-3-oxide hexafluorophosphate (HATU) as coupling agent [34]. Sequential addition of HATU (1.5 eqs) in the presence of N, N-diisopropylethylamine DIPEA (1.5 eq) to DAICA in DMF under controlled atmosphere resulted in the activation of the carboxylic group which was eventually converted to the desired amide further to addition of the amine (Figure 1). To optimize the reaction parameters, the process was followed by HPLC analysis. At 15 min reaction time, the peak corresponding to DAICA (t_R_ = 5.5 min) completely disappeared, while the formation of a new component eluted at 9.8 min, likely attributable to the species formed by reaction of HATU with DAICA (**A**) was maximal (Figure S1). At this time point, addition of 1-butanamine (1.5 eqs) led to the disappearance of the peak at 9.8 min with concomitant formation of a product eluted at 8.1 min, identified as DAICA butanamide (LCMS evidence) (Figure S2).

The DHICA amide was straightforwardly recovered by precipitation thanks to simple addition of water to the reaction mixture. The precipitate that separated was filtered and washed sequentially with water and 0.01 M HCl to remove excess HATU/its N-oxide and the unreacted amine, respectively. The product obtained in 85% yield proved pure on HPLC (Figure S3) and LC-MS analysis and was subjected to ^1^H and ^13^C NMR characterization (Figures S4 and S5 and Table S1).

The same procedure was then extended to other selected amines, including aniline and two diamines i.e., 1,4-diaminebutane and 1,6-diaminehexane. All products were obtained in satisfactory pure form without the need of purification steps and characterized by ^1^H NMR and ^13^C NMR (Figures S6–S11, Tables S2–S4). The yields obtained for the acetylated carboxamides are reported in Table 1. The amides were stored in the stable *O*-acetylated form and hydrolyzed just before use.

Removal of the acetyl group from DAICA amides was carried out under controlled conditions to avoid product oxidation. The compound dissolved in dichlorometane/methanol 9:1 *v*/*v* at 8 mM concentration and taken under an inert atmosphere was treated with alcoholic 3 M KOH. Deprotection was complete after 5 min as assessed by HPLC analysis (Figure S12), and the mixture was then acidified to pH 2 with aqueous acids. The product was recovered after work up of the mixture in almost quantitative yields. Purity of the compounds was also checked by ^1^H-NMR analysis (see Figure S13 for a representative example).

**Figure 2:**
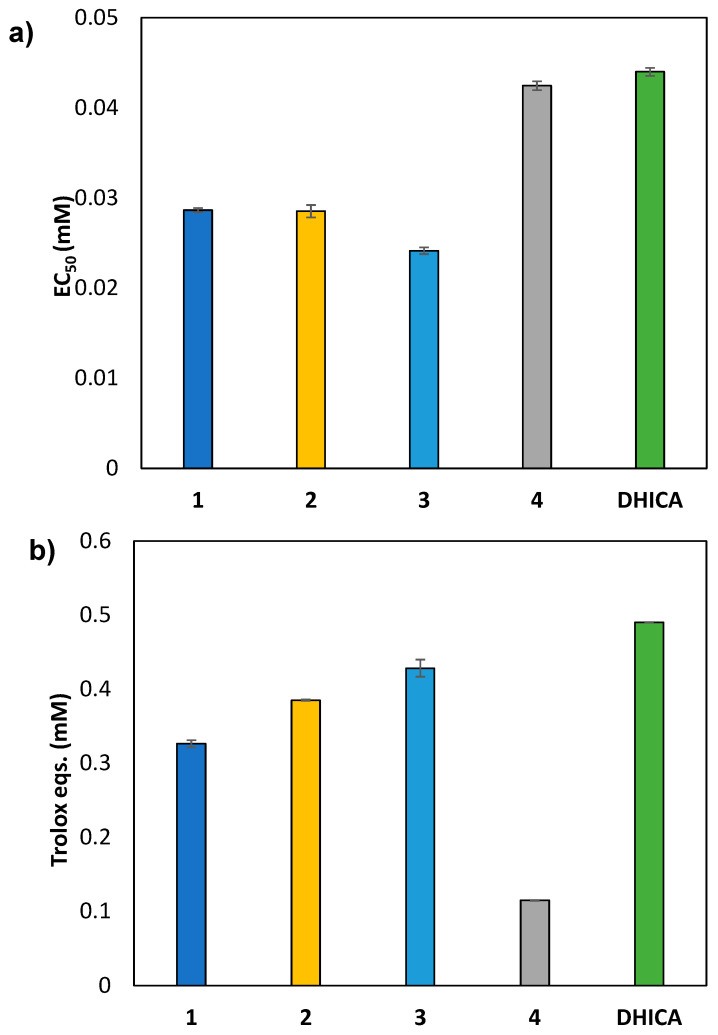
Antioxidant properties of DHICA carboxamides. (**a**) DPPH assay; (**b**) FRAP assay. Reported are the mean ± SD values of at least three experiments.

After deprotection of the catechol group, the antioxidant potential of the carboxamides was evaluated by two widely used chemical tests, namely the 2,2-diphenyl-1-picrylhydrazyl (DPPH) assay measuring the H-donor ability of a given species, and the ferric reducing antioxidant power (FRAP) test as a measure of the Fe (III) reducing power. Figure 2a shows data obtained in the DPPH assay expressed as EC_50_ values. Compounds **1**–**3** showed higher antioxidant activity compared to DHICA, while compound **4** was substantially comparable. Figure 2b shows data obtained in the FRAP assay expressed as Trolox eqs.

Carboxamides **1**–**3** exhibited a good reducing power, while **4** showed a modest but appreciable activity. In all cases, the activity in the FRAP test was lower than that of DHICA likely as a result of the lower solubility of the compounds in the assay solution medium, i.e., acid aqueous solution.

Overall, these results further add to the applications of DHICA carboxamides synthesized indicating a potential photoprotective action as a result of their ability to control the level of reactive oxygen species generated in vivo on solar exposure and the associated oxidation processes.

### 2.2. Oxidation of DHICA Amides: Pigment Formation and Properties Characterization

**Figure 3:**
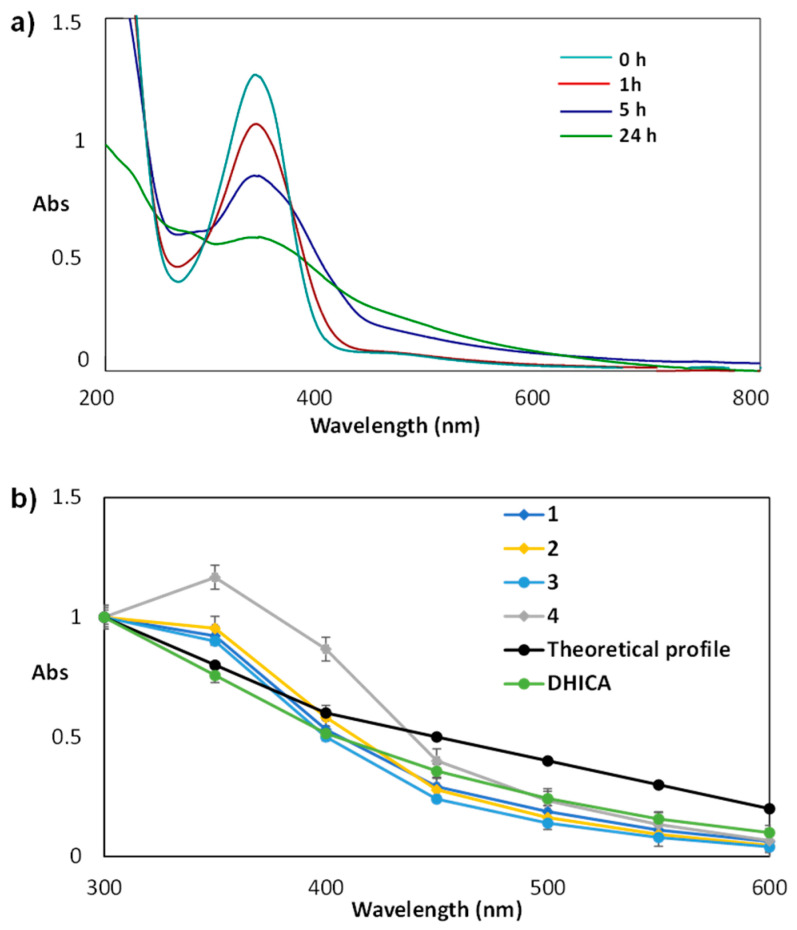
(**a**) UV–Vis spectrum of the course of the aerial oxidation of **1** over time; (**b**) relative absorbance of the oxidation mixtures of all the synthesized amides and DHICA at 24 h reaction time against the theoretical monotonic profile. Reported are the mean ± SD values of at least three experiments. (Values normalized against the absorbance at 300 nm for each mixture).

For all DHICA carboxamides, oxidation was carried out in air under slightly alkaline conditions (50 mM carbonate buffer at pH 9.0) over 24 h. Pigment formation and consumption of the substrate was evaluated by UV–Vis spectrophotometry (Figure S14). As the oxidation progresses, the absorption peak of the starting product at 320 nm decreases and is converted in a broad absorption covering the UVA and visible region as shown in Figure 2a for **1** as a representative example. After 24 h, separation of a melanin-like pigmented material is observed (Figure 3a).

Shown in Figure 3b is the absorbance over the UV–Vis spectrum obtained at 24 h for all the amides normalized against the absorbance at 300 nm. All of them show a profile similar to that of DHICA indicating that the pigments formed may indeed be taken as true melanins. Comparison with the monotonic profile expected for an ideal melanin pigment indicates higher absorbance in the UVA region for all the samples except for DHICA, as particularly evident for compound **4,** exhibiting a significant absorption in the 350–450 nm region.

**Figure 4:**
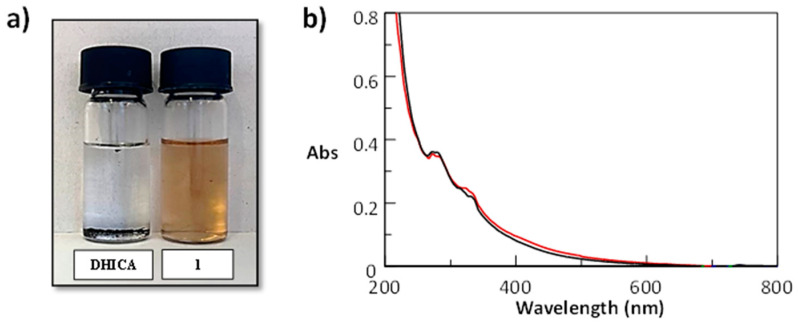
(**a**) Ethanolic solution (3 mg/mL) of the pigment from DHICA carboxybutanamide **1** (right) in comparison with DHICA melanin (left); *(***b**) UV–Vis spectrum prior and after filtration of the ethanolic solution of the pigment from **1** (1 mg/mL).

The pigments from oxidation of DHICA amides were prepared on hundreds of milligrams scale, recovered from the reaction mixtures, extensively washed, and dried. The solubility properties were tested using ethanol chosen as a most relevant solvent for dermocosmetic applications. The solution obtained by dissolving 1 mg/mL of the pigment from **1** is shown in Figure 3a in comparison with that obtained for DHICA melanin that proves completely insoluble at these *w*/*v* values. The ethanolic solutions of the pigments from all the synthesized amides appear brown in color on account of their intense absorption in the visible region. (Figure S15). The solubility of the pigments was checked by recording the UV–Vis spectrum prior and after filtration through a 25-micron polyamide membrane (Figure 4b and Figure S16).

## 3. Materials and Methods

3,4-dihydroxy-L-phenylalanine (L-DOPA), potassium ferricyanide, sodium bicarbonate, sodium dithionite, *N*,*N*-diisopropylethylamine (DIPEA), anhydrous dimethylformamide (DMF), 1-[bis(dimethylamino)methylene]-1*H*-1,2,3-triazolo[4,5-b]pyridinium-3-oxide hexafluorophosphate (HATU), 1,4-butanediamine, 1,6-hexanediamine, aniline, 1-butanamine, pyridine (Py), and acetic anhydride were purchased from Sigma-Aldrich (Milan, Italy).

^1^H and ^13^C NMR spectra were recorded in DMSO-d_6_ at 400 MHz on a Bruker spectrometer (Milan, Italy). Chemical shifts are given in ppm. Proton and carbon resonance assignments (see Figures S4–S11) were based on 2D NMR analysis (^1^H, ^1^H COSY, ^1^H,^13^C HSQC, ^1^H,^13^C HMBC, not shown).

The UV–Vis spectra were recorded on a Jasco V-730 spectrophotometer (Cernusco sul Naviglio, Milan, Italy).

HPLC analyses were performed on an Agilent 1100 binary pump instrument equipped with a Shimadzu SPD-10AV VP UV–visible detector (Agilent, G1314A, Cernusco sul Naviglio, Milan, Italy) using an octadecylsilane-coated column, 250 mm × 4.6 mm, 5 μm particle size (Phenomenex Sphereclone ODS- Castel Maggiore, Bologna, Italy) at 0.7 mL/min. Detection wavelength was set at 300 nm.

LC/MS analyses were performed in positive ion mode using an Agilent 1260/6230DA ESI-TOF instrument (Cernusco sul Naviglio, Milan, Italy) under the following conditions: 35 psig nebulizer pressure; drying gas (nitrogen) flushed at 5 L/min at a temperature of 325 °C; capillary voltage 3500 V; fragmentor voltage 175 V. An Eclipse Plus C18 column (Agilent, Cernusco sul Naviglio, Milan, Italy) (150 × 4.6 mm, 5 µm) and 0.1% formic acid-methanol 40:60 *v*/*v* as the eluant at a flow rate of 0.4 mL/min was used.

### 3.1. Synthesis of DHICA and DAICA

DHICA was prepared as described before by ferricyanide oxidation of DOPA. [32] For the synthesis of DAICA, DHICA (700 mg) was acetylated with acetic anhydride (7 mL) and pyridine (350 µL) for 24 h, followed by hydrolysis with a 1:1 *v*/*v* water-methanol mixture at 90 °C for 2 h. After removal of the volatile components at a rotary evaporator, a powder lightly yellow in color was obtained (98% yield) [32]. Purity of the compound was checked by ^1^H NMR, LC-MS, and UV–Vis [35] analysis.

### 3.2. Synthesis of DAICA Carboxamides: General Procedure

A mixture of DAICA (281 mg), HATU (1.5 eqs, 759 mg), and DIPEA (2 eqs, 474.5 µL) in anhydrous DMF (5.62 mL) was stirred under an argon atmosphere for 15 min. The proper amine, i.e., 1-butanamine (202 µL, 1.5 eqs), 1,4-diamminobutane (152 µL, 1.5 eqs), 1,6-diamminohexane (176 mg, 1.5 eqs), or aniline (138 µL, 1.5 eqs) was then added, and the mixture was stirred for an additional 30 min at 25 °C. The course of the reaction was followed by HPLC analysis of aliquots withdrawn from the mixture after 1:1000 dilution in methanol. The time point for amine addition and for complete product formation was chosen based on HPLC analysis. At reaction completion, addition of distilled water resulted in separation of a precipitate that was recovered by filtration and sequentially washed with water (3 times) and 0.01 M HCl (2 times). The product thus obtained (yields reported in Table 1) was analyzed for purity by LC-MS and ^1^H NMR.

*N*-butane-5,6-diacetoxy-1*H*-indole-2-carboxamide (**1**)

ES-MS: *m*/*z* 333 (M+H)^+^

^1^H-NMR (DMSO-d_6_): δ (ppm): 0.90 (3H, t, J = 7.2 Hz), 1.33 (2H, sext, J = 7.2 Hz), 1.51 (2H, quint, J = 7.6 Hz), 2.25 (3H, s), 2.26 (3H, s), 3.21 (2H, m), 7.14 (1H, dd, J = 2.0, 0.8 Hz), 7.21 (1H, d, J = 0.8 Hz), 7.45 (1H, bs) 8.47 (1H, t, J = 5.8 Hz), 11.74 (1H, bs)

^13^C-NMR (DMSO-d_6_): δ (ppm): 14.3 (CH_3_), 20.1 (CH_2_), 20.8 (CH_3_), 20.9 (CH_3_), 31.8 (CH_2_), 39.3 (CH_2_), 102.8 (CH), 106.7 (CH), 115.2 (CH), 124.9 (C), 133.9 (C) 134.0 (C), 136.9 (C), 139.5 (C), 161.0 (CONH), 169.2 (COOCH_3_), 169.3 (COOCH_3_)

Deprotection of DAICA carboxybutanamide was carried out by dissolving the compound (314 mg, 1.2 mmol) in dichlorometane/methanol 9:1 *v*/*v* (final concentration 3.6 mM); the solution was placed under an argon atmosphere and a 0.2 M KOH solution in methanol that has been previously purged with argon was added. The mixture was stirred for 15 min, acidified to pH 3, and brought to dryness. A yellow solid was obtained together with the salts. The residue was dissolved with water at pH 3 and extracted three times with ethyl acetate. The organic layers were brought to dryness and the product used without further purification (75% yield). HPLC and ^1^H-NMR analysis confirmed a substantial purity of the compound (Figures S12 and S13). The same hydrolytic procedure was applied to the other carboxamides synthesized.

^1^H-NMR of deacetylated **1** (DMSO-d_6_): δ (ppm): 0.89 (3H, t, J = 7.2 Hz), 1.32 (2H, sext, J = 7.2 Hz), 1.48 (2H, quint, J = 7.2 Hz), 3.28 (2H, m), 6.77 (1H, bs), 6.81 (1H, bs), 6.84 (1H, bs), 8.22 (1H, t, J = 6 Hz,), 10.95 (1H, bs)

*N*,*N*′-(Butane-1,4-diyl)bis(5,6-diacetoxy-1*H*-indole-2-carboxamide) (**2**)

ES-MS: *m*/*z* 607 (M+H)^+^

^1^H-NMR (DMSO-d_6_): δ (ppm): 1.58 (4H, t, J = 8.0 Hz), 2.25 (3Hx2, s), 2.26 (3Hx2, s), 3.32 (4H, m), 7.12 (1Hx2, dd, J = 2.4, 0.8 Hz), 7.22 (1Hx2, dd, J = 0.8 Hz), 7.45 (1Hx2, bs) 8.51 (2H, t, J = 6 Hz), 11.75 (1Hx2, bs)

^13^C-NMR (DMSO-d_6_): δ (ppm): 20.8 (CH_3_), 20.9 (CH_3_), 27.2 (CH_2_), 39.8 (CH_2_), 102.8 (CH), 106.7 (CH), 115.2 (CH), 124.9 (C), 133.9 (C) 134.0 (C), 136.9 (C), 139.5 (C), 161.0 (CONH), 169.2 (COOCH_3_), 169.4 (COOCH_3_).

*N,N*′-(Hexane-1,6-diyl)bis(5,6-dihydroxy-1*H*-indole-2-carboxamide) (**3**)

ES-MS: *m*/*z* 635 (M+H)^+^

^1^H-NMR (DMSO-d_6_): δ (ppm): 1.37 (4H, m), 1.56 (4H, m), 2.25 (3Hx2, s), 2.26 (3Hx2, s), 3.32 (4H, m), 7.12 (1Hx2, bs), 7.22 (1Hx2, bs), 7.45 (1Hx2, s) 8.51 (2H, t, J = 6 Hz), 11.72 (1Hx2, bs)

^13^C-NMR (DMSO-d_6_): δ (ppm): 20.8 (CH_3_), 20.9 (CH_3_), 26.7 (CH_2_), 29.6 (CH_2_), 39.3 (CH_2_), 102.8 (CH), 106.5 (CH), 115.2 (CH), 124.9 (C), 133.9 (C), 134.0 (C), 136.8 (C), 139.3 (C), 161.0 (CONH), 169.2 (COOCH_3_) 169.4 (COOCH_3_).

5,6-diacetoxy-*N*-phenyl-1*H*-indole-2-carboxamide (**4**)

ES-MS: *m*/*z* 353 (M+H)^+^

^1^H-NMR (DMSO-d_6_): δ (ppm): 2.27 (3H, s), 2.28 (3H, s), 7.10 (1H, t, J = 8 Hz), 7.27 (1H, t, J = 0.8), 7.37 (2H, t, J = 8.0 Hz), 7.44 (1H, dd, J = 2.4, 0.8), 7.55 (1H, bs), 7.88 (2H, d, J = 2.0 Hz), 10.24 (s, 1H), 11.92 (s, 1H)

^13^C-NMR (DMSO-d_6_): δ (ppm): 20.8 (CH_3_), 20.9 (CH_3_), 104.4 (CH), 106.8 (CH), 115.4 (CH), 120.6 (2xCH), 124.5 (C), 124.1 (1xCH) 129.2 (2xCH), 133.4 (C), 134.5 (C), 137.1 (C), 139.3 (C), 140.0 (C), 159.9 (CONH), 169.2 (COOCH_3_), 169.4 (COOCH_3_)

### 3.3. DPPH Assay 

To a 0.2 mM ethanolic solution of DPPH, DHICA carboxamides or DHICA were added at a final dose in the range 0.010–0.055 mM. After 10 min under stirring at room temperature, the absorbance of the solution at 515 nm was measured. Experiments were run in triplicate [36].

### 3.4. Ferric Reducing/Antioxidant Power (FRAP) Assay 

To 0.3 M acetate buffer (pH 3.6) containing 1.7 mM FeCl_3_ and 0.83 mM TPTZ, DHICA carboxamides or DHICA were added at a final dose in the range 0.0025–0.0185 mM. After 10 min under stirring at room temperature, the absorbance of the solution at 593 nm was measured. [37]

### 3.5. Preparation of Melanin from DHICA and DHICA Carboxamides

Melanins were prepared by aerobic oxidation of DHICA or deactylated DHICA carboxamides (1 mM) in 0.05 M carbonate buffer at pH 9. After 24 h, the precipitate was collected by acidification to pH 3 and centrifugation (7000 rpm, 10 min, 4 °C). The sample was washed three times with 0.01 M HCl (15 mL) and lyophilized. Melanins from DHICA and DHICA carboxamides were obtained in average 85 and 95 *w*/*w* yield, respectively.

### 3.6. Solubility Test of Melanins

Melanins from DHICA or DHICA carboxamides were added to ethanol at 1 mg/mL and the resulting mixtures were taken under stirring for 15 min. The solubility of the pigments was checked by running the UV–Vis spectrum prior and after filtration of the solution through a 25-micron polyamide membrane.

## 4. Conclusions

Manipulation of the molecular scaffold of catechol systems is a currently pursued synthetic target in view of the variety of chemical facets of these compounds that make them valuable tools for the most diverse applications. In the present paper, we optimized a procedure for preparation of carboxamides of DHICA, the main biosynthetic precursor of melanin pigments with the aim of gaining access to pigments with more favorable properties with respect to those of the natural pigments. Satisfactorily high yields (>85%) were obtained for all amides that could be recovered from the reaction mixture in enough pure form not requiring purification steps. The procedure was probed with a representative aliphatic and aromatic amine as well as with diamines of different chain length. The the synthesized carboxamides showed an antioxidant activity comparable or even superior to that of DHICA which has been shown as a powerful antioxidant metabolite [38]. Though safety evaluation of the carboxamides **1**–**4** is still to be assessed and represents a goal of future investigation, the lack of toxicity of structurally related carboxamides is encouraging along this direction [39].

The melanin-like pigmented materials obtained by oxidative polymerization of the synthesized amides showed chromophoric features similar to those of the DHICA-derived pigment but exhibited higher absorption in the UVA region and good solubility in alcoholic solvents, a feature of great interest for the exploitation of these materials as ingredients of dermocosmetic formulations. Assay of the properties of the DHICA carboxamides-derived pigments that may be relevant for this use represents a goal of future investigation.

Peculiar properties deserving further investigation may also be expected for the pigment obtained from the *N*-phenyl carboxamide for which π-π stacking interactions of the phenyl rings may stabilize specific arrangements of the polymeric systems as suggested by the intense chromophore in the visible region. On the other hand, the dicarboxamides of DHICA were designed to access to melanin-like pigments that, differently from natural pigments, might exhibit a higher degree of regiocontrol and possibly give rise to supramolecular arrangements determining unexpected properties for applications in material science.

In sum, the availability of a variety of DHICA carboxamides provides new research stimuli from either the application or the academic perspective.

## Figures and Tables

**Table 1:** Structure and yields of the synthesized DAICA carboxamides.

Amide Derivatives of DAICA	Yield (%)
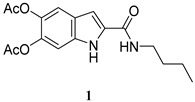	85
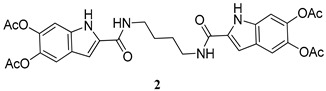	90
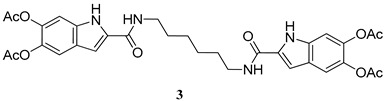	87
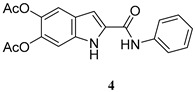	85

## Data Availability

Not applicable.

## Supplementary Materials

The following supporting information can be downloaded at: www.mdpi.com/article/10.3390/molecules27154816/s1, Figure S1: HPLC profile of the amidation reaction of DAICA at 10 min after addition of HATU (λ = 300 nm). Figure S2: HPLC profile of the course of the amidation reaction at 5 min after addition of 1-butanamine (λ = 300 nm). Figure S3: HPLC profile of DAICA butanamide after purification (λ = 300 nm). Figure S4: ^1^H NMR spectrum of N-butane-5,6-diacetoxy-1H-indole-2-carboxamide (1) (DMSO-d_6_). Figure S5: ^13^C NMR spectrum of N-butane-5,6-diacetoxy-1H-indole-2-carboxamide (1) (DMSO-d_6_). Table S1: NMR spectral data (ppm) for 1. Resonance assignment based on 2D NMR analysis. Figure S6: ^1^H NMR spectrum N,N′-(butane-1,4-diyl)bis(5,6-diacetoxy-1H-indole-2-carboxamide) (2) (DMSO-d_6_). Figure S7: ^13^C NMR spectrum N,N′-(butane-1,4-diyl)bis(5,6-diacetoxy-1H-indole-2-carboxamide) (2) (DMSO-d_6_). Table S2: NMR spectral data (ppm) for 2. Resonance assignment based on 2D NMR analysis. Figure S8: ^1^H NMR spectrum of N,N′-(hexane-1,6-diyl)bis(5,6-diacetoxy-1H-indole-2-carboxamide) (3) (DMSO-d_6_). Figure S9: ^13^C NMR spectrum of N,N′-(hexane-1,6-diyl)bis(5,6-diacetoxy-1H-indole-2-carboxamide) (3) (DMSO-d_6_). Table S3: NMR spectral data (ppm) for 3. Resonance assignment based on 2D NMR analysis. Figure S10: ^1^H NMR spectrum of 5,6-diacetoxy-N-phenyl-1H-indole-2-carboxamide of DHICA (4) (DMSO-d_6_). Figure S11: ^13^C NMR spectrum of 5,6-diacetoxy-N-phenyl-1H-indole-2-carboxamide of DHICA (4) (DMSO-d_6_). Table S4: NMR spectral data (ppm) for 4. Resonance assignment based on 2D NMR analysis. Figure S12: Chromatogram of deacetylated 1 (λ = 300 nm). Figure S13: ^1^H NMR spectrum of deacetylated 1 (DMSO-d_6_). Figure S14: UV-Vis spectrophotometric monitoring of the course of the aerial oxidation of DHICA carboxamides over time. Figure S15: Digital photos of pigments as obtained from DHICA carboxamides in carbonate buffer at pH 9 after 24 h under stirring. Figure S16: UV-vis spectrum prior and after filtration of the ethanol solution (1 mg/mL) of carboxamides and DHICA.

## Sample Availability

Samples of the compounds **1**–**4** are available from the authors.
