# Supplementary material for: A Straightforward Access to New Amides of the Melanin Precursor 5,6-Dihydroxyindole-2-carboxylic Acid and Characterization of the Properties of the Pigments Thereof
Source: Molecules. 2022 Jul 27;27(15):4816. doi: 10.3390/molecules27154816 (PMC9369804; doi:10.3390/molecules27154816)
Supplement: Supplementary file 1 [file molecules-27-04816-s001.zip › molecules-1814567-supplementary.pdf]

Supporting information

A straightforward access to new amides of the melanin precursor 5,6-dihydroxyindole-2-carboxylic acid and characterization of the properties of the pigments thereof

Table of contents	
**Figure S1.** HPLC profile of the amidation reaction of DAICA at 10 min after addition of HATU (λ = 300 nm).	p. 1
**Figure S2.** HPLC profile of the course of the amidation reaction at 5 minutes after addition of 1-butanamine (λ = 300 nm).	p. 2
**Figure S3.** HPLC profile of DAICA butanamide after purification (λ = 300 nm).	p. 3
**Figure S4**. ^{1}H NMR spectrum of *N*-butane-5,6-diacetoxy-1*H*-indole-2-carboxamide (**1**) (DMSO-d_6).	p. 4
**Figure S5**. ^{13}C NMR spectrum of *N*-butane-5,6-diacetoxy-1*H*-indole-2-carboxamide (**1**) (DMSO-d_6).	p. 5
**Table S1.** NMR spectral data (ppm) for **1.** Resonance assignment based on 2D NMR analysis	p. 6
**Figure S6**. ^{1}H NMR spectrum *N,N'*-(butane-1,4-diyl)bis(5,6-diacetoxy-1*H*-indole-2-carboxamide) (**2**) (DMSO-d_6).	p. 7
**Figure S7**. ^{13}C NMR spectrum *N,N'*-(butane-1,4-diyl)bis(5,6-diacetoxy-1*H*-indole-2-carboxamide) (**2**) (DMSO-d_6).	p. 8
**Table S2.** NMR spectral data (ppm) for **2.** Resonance assignment based on 2D NMR analysis	p. 9
**Figure S8**. ^{1}H NMR spectrum of *N,N'*-(hexane-1,6-diyl)bis(5,6-diacetoxy-1*H*-indole-2-carboxamide) (**3**) (DMSO-d_6).	p. 10
**Figure S9**. ^{13}C NMR spectrum of *N,N'*-(hexane-1,6-diyl)bis(5,6-diacetoxy-1*H*-indole-2-carboxamide) (**3**) (DMSO-d_6).	p. 11
**Table S3.** NMR spectral data (ppm) for **3.** Resonance assignment based on 2D NMR analysis	p. 12
**Figure S10**. ^{1}H NMR spectrum of 5,6-diacetoxy-*N*-phenyl-1*H*-indole-2-carboxamide of DHICA (**4**) (DMSO-d_6).	p. 13
**Figure S11**. ^{13}C NMR spectrum of 5,6-diacetoxy-*N*-phenyl-1*H*-indole-2-carboxamide of DHICA (**4**) (DMSO-d_6).	p. 14
**Table S4.** NMR spectral data (ppm) for **4.** Resonance assignment based on 2D NMR analysis	p. 15
**Figure S12.** Chromatogram of deacetylated **1** (λ = 300 nm).	p. 16
**Figure S13**. ^{1}H NMR spectrum of deacetylated **1** (DMSO-d_6).	p. 17
**Figure S14**. UV-Vis spectrophotometric monitoring of the course of the aerial oxidation of DHICA carboxamides over time.	p. 18
**Figure S15**. Digital photos of pigments as obtained from DHICA carboxamides in carbonate buffer at pH 9 after 24 h under stirring.	p. 19
**Figure S16.** UV-vis spectrum prior and after filtration of the ethanol solution (1 mg/mL) of carboxamides and DHICA.	p. 20

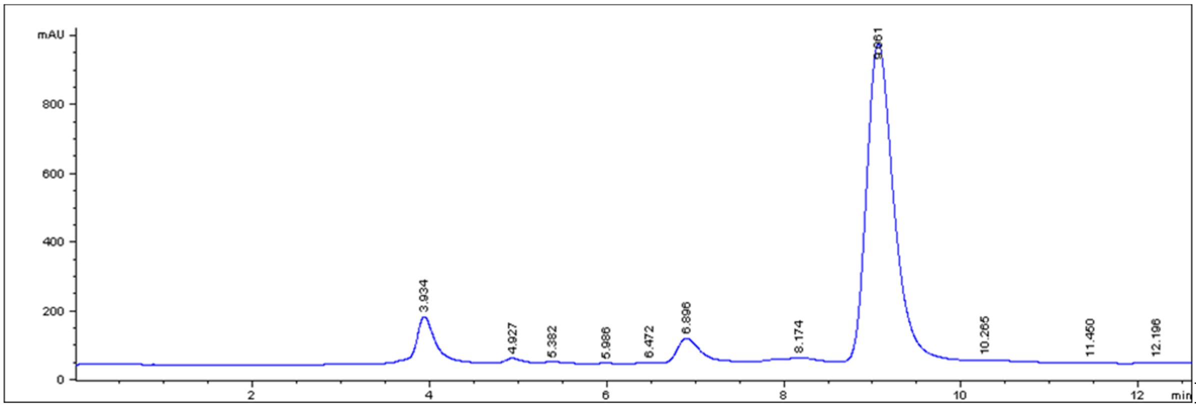

Figure S1. HPLC profile of the amidation reaction of DAICA at 10 min after addition of HATU (λ = 300 nm).

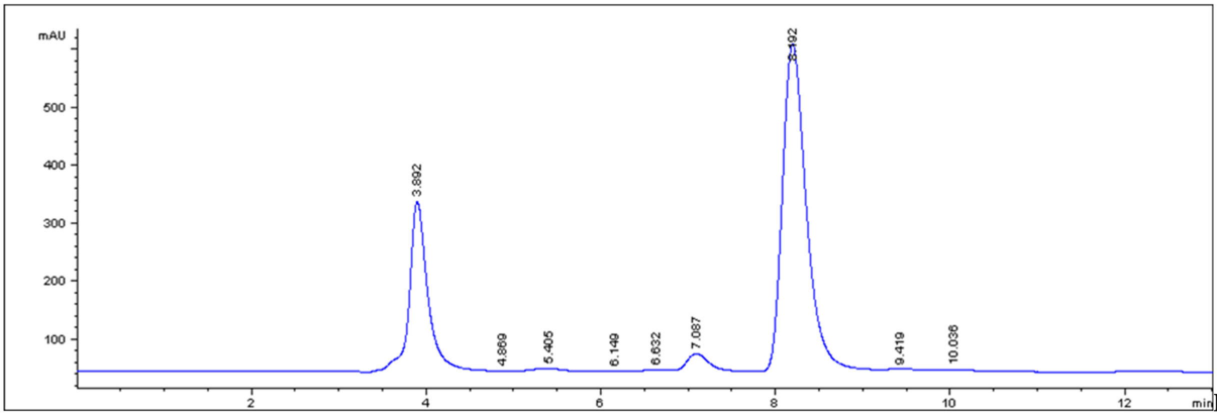

Figure S2. Chromatogram of the course of the amidation reaction at 5 minutes after addition of 1-butanamine (λ = 300 nm).

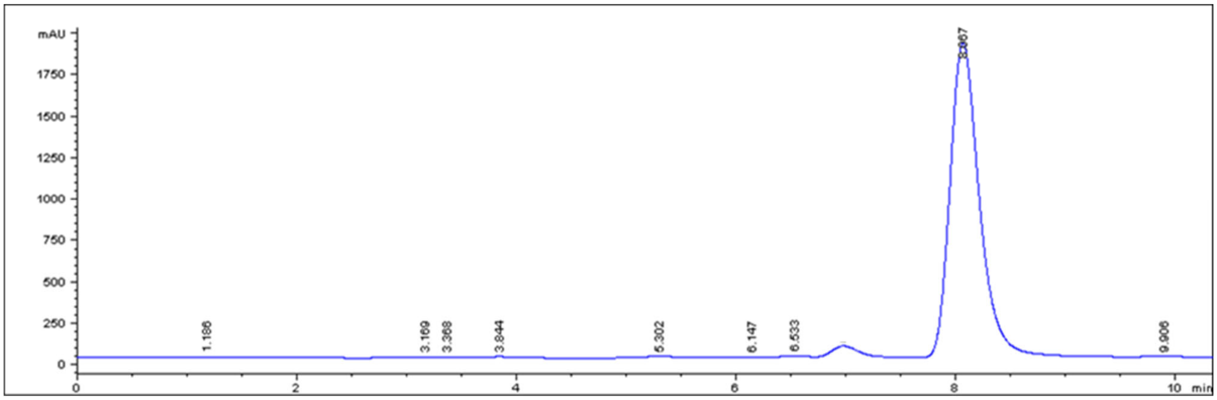

Figure S3. HPLC profile of DAICA butanamide after purification (λ = 300 nm).

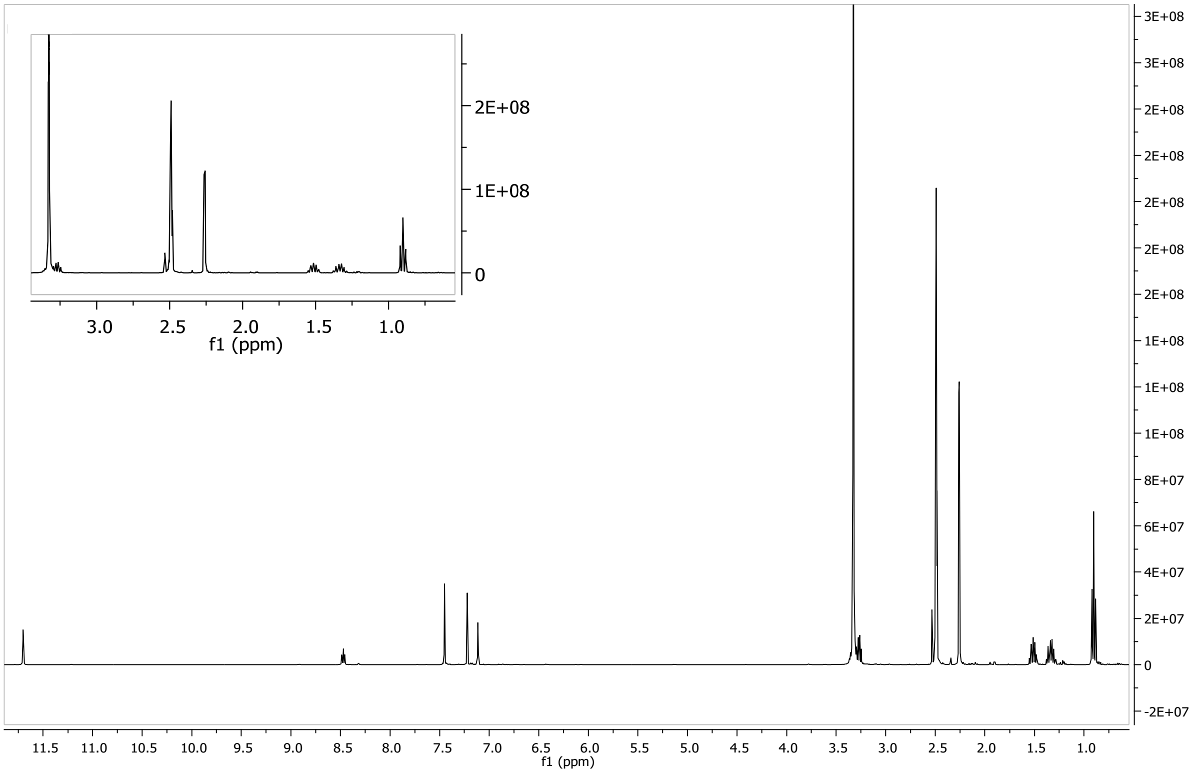

Figure S4. ^{1}H NMR spectrum of *N*-butane-5,6-diacetoxy-1*H*-indole-2-carboxamide (**1**) (DMSO-d_6).

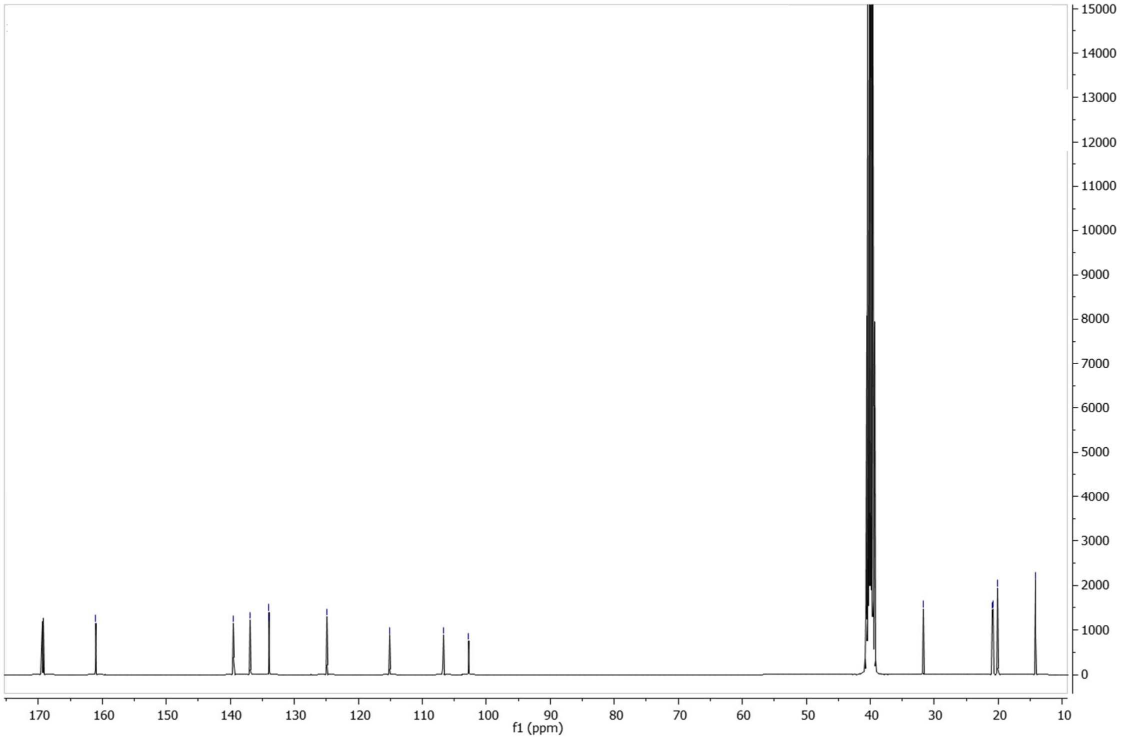

Figure S5. ^{13}C NMR spectrum of *N*-butane-5,6-diacetoxy-1*H*-indole-2-carboxamide (**1**) (DMSO-d_6).

Table S1. NMR spectral data (ppm) for **1***.

	^{1}H (J, Hz)	^{13}C
**CH₃**CH₂CH₂CH₂NH	0.90(t,7.2)	14.3
CH₃**CH₂**CH₂CH₂NH	1.33(s,7.2)	20.1
CH₃CH₂**CH₂**CH₂NH	1.51(quint, 7.6)	31.8
**CH₃**COO-5 indole	2.25(s)	20.8
**CH₃**COO-6 indole	2.26(s)	20.9
CH₃CH₂CH₂**CH₂**NH	3.21(m)	39.3
**CH-3** indole	7.14(dd, 0.8, 2.0)	102.8
**CH-4** indole	7.45(d, 0.8)	106.7
**CH-7** indole	7.21(bs)	115.2
CH₂**NH**CO	8.47(t,5.8)	
**NH** indole	11.74(bs)	
**C9** indole		124.9
**C2** indole		133.9
**C8** indole		134.0
**C6** indole		136.9
**C5** indole		139.5
NH**C**O		161.0
CH₃**C**OO-5 indole		169.2
CH₃**C**OO-6 indole		169.3

*Resonance assignment based on 2D NMR spectra (not shown).

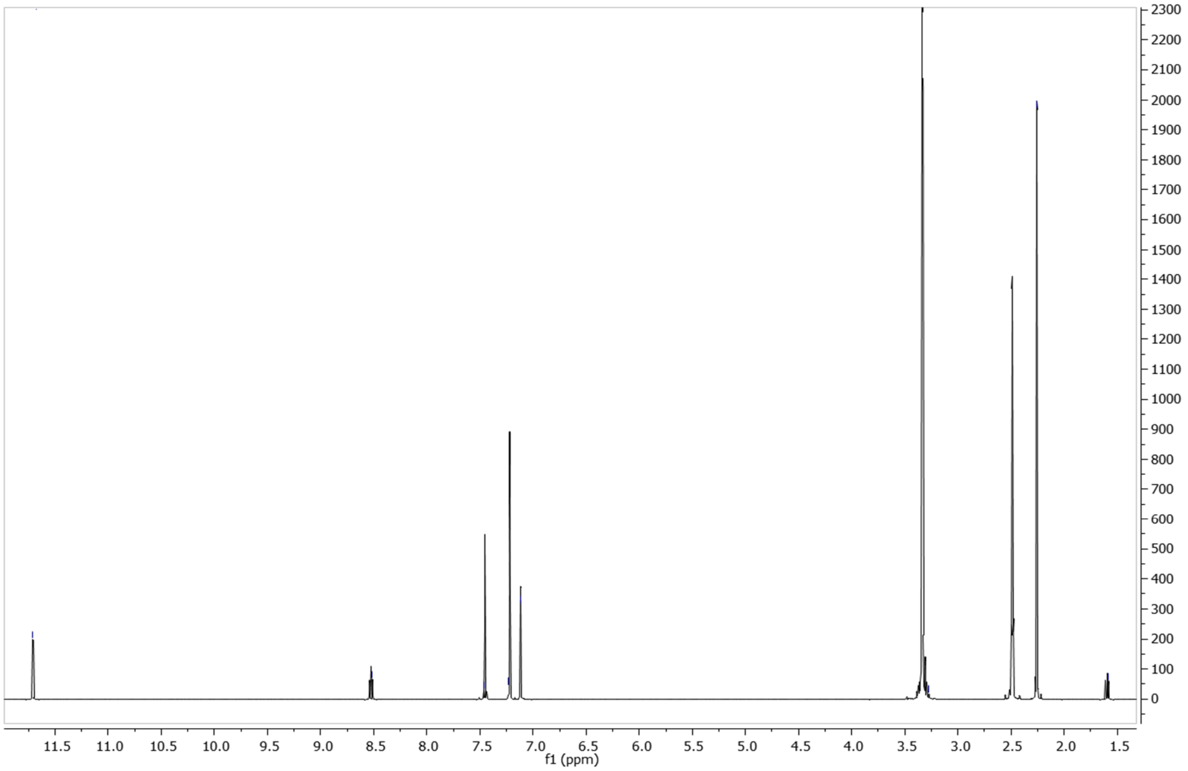

Figure S6. ^{1}H NMR spectrum *N,N′*-(butane-1,4-diyl)bis(5,6-diacetoxy-1*H*-indole-2-carboxamide) (**2**) (DMSO-d$_6$).

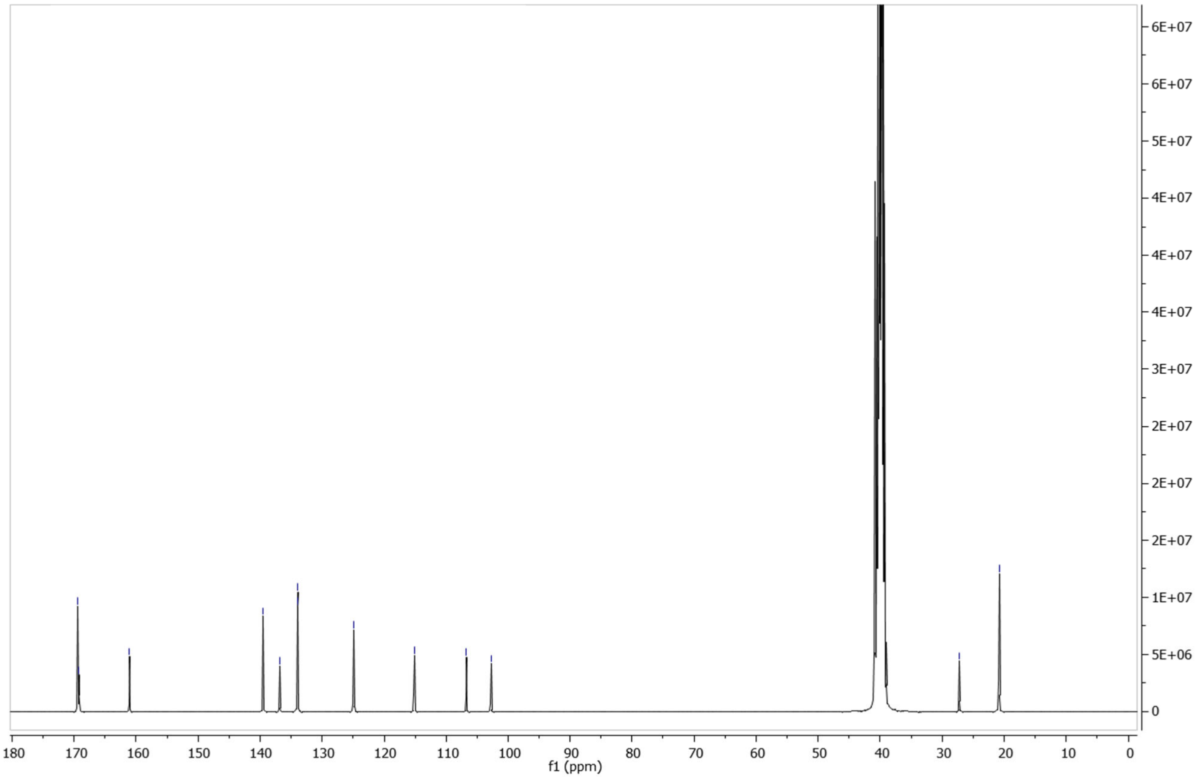

Figure S7. ^{13}C NMR spectrum *N,N′*-(butane-1,4-diyl)bis(5,6-diacetoxy-1*H*-indole-2-carboxamide) (**2**) (DMSO-d$_6$).

Table S2. NMR spectral data (ppm) for **2.**

	^{1}H (J, Hz)	^{13}C
NHCH$_2$**CH$_2$**CH$_2$CH$_2$NH	1.58(t,8.0)	27.2
**CH$_3$**COO-5 indole	2.25(s)	20.8
**CH$_3$**COO-6 indole	2.26(s)	20.9
NH**CH$_2$**CH$_2$CH$_2$CH$_2$NH	3.32(m)	39.8
**CH-3** indole	7.12(dd, 0.8, 2.4)	102.8
**CH-4** indole	7.45(d, 0.8)	106.7
**CH-7** indole	7.21(bs)	115.2
CH$_2$**NH**CO	8.51(t,6.0)	
**NH** indole	11.72(bs)	
**C9** indole		124.9
**C2** indole		133.9
**C8** indole		134.0
**C6** indole		136.9
**C5** indole		139.5
NH**C**O		161.0
CH$_3$**C**OO-5 indole		169.2
CH$_3$**C**OO-6 indole		169.4

*Resonance assignment based on 2D NMR spectra (not shown)

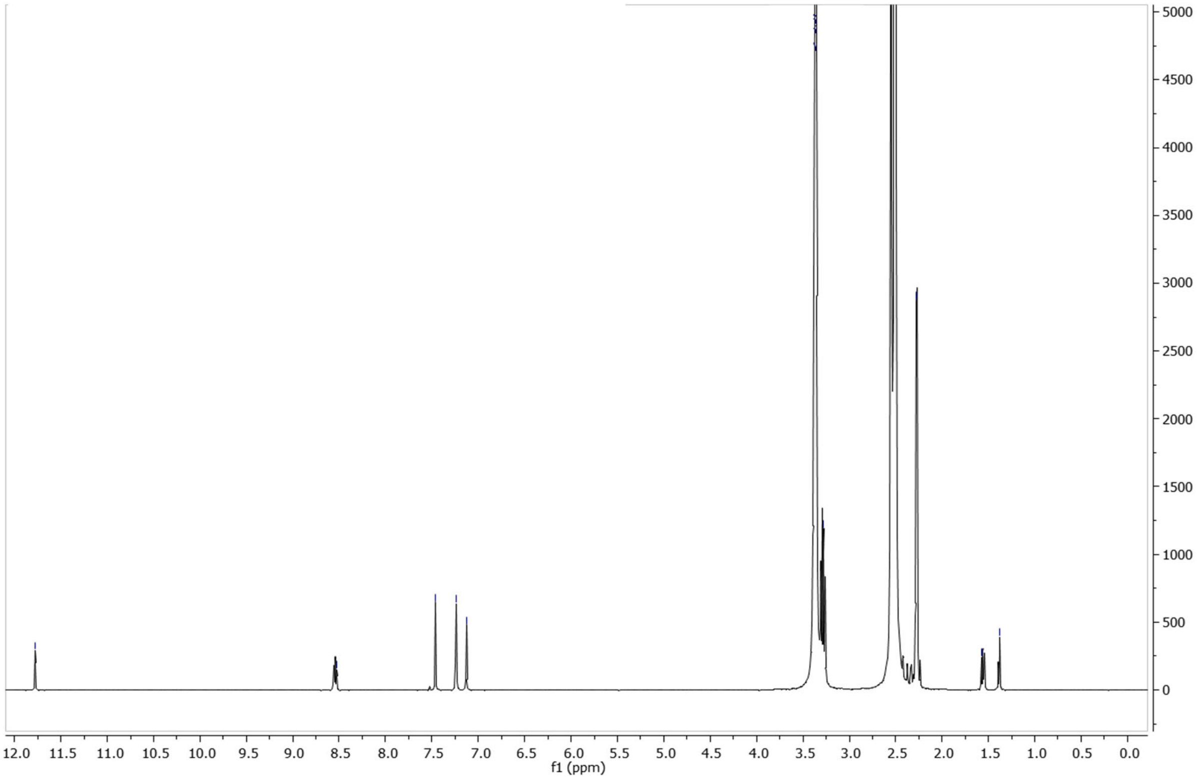

Figure S8. ^{1}H NMR spectrum of *N,N′*-(hexane-1,6-diyl)bis(5,6-diacetoxy-1*H*-indole-2-carboxamide) (**3**) (DMSO-d$_6$).

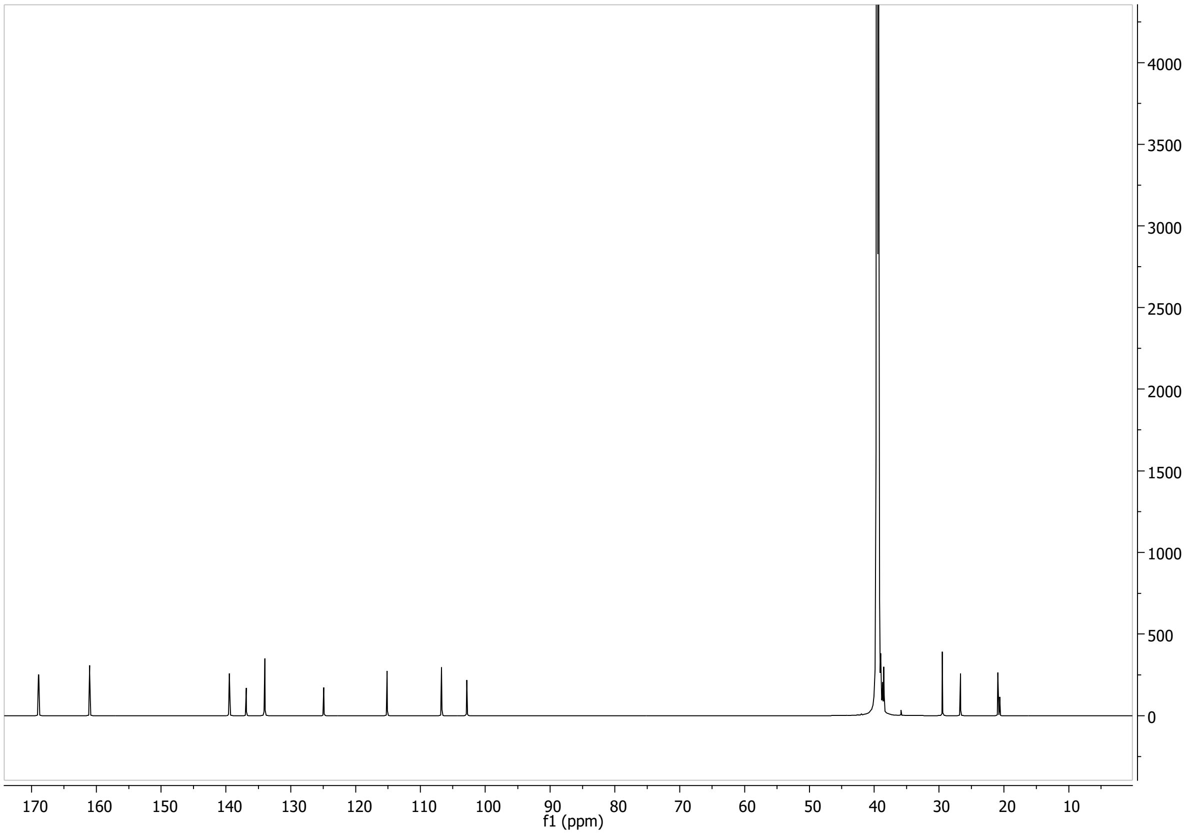

Figure S9. ^{13}C NMR spectrum of *N,N′*-(hexane-1,6-diyl)bis(5,6-diacetoxy-1*H*-indole-2-carboxamide) **(3)** (DMSO-d_6).

Table S3. NMR spectral data (ppm) for **3.**

	**^1H (J, Hz)**	**^{13}C**
NHCH$_2$CH$_2$**CH$_2$**CH$_2$CH$_2$CH$_2$NH	1.37(m)	26.7
NHCH$_2$**CH$_2$**CH$_2$CH$_2$CH$_2$CH$_2$NH	1.56(m)	29.6
**CH$_3$**COO-5 indole	2.25(s)	20.8
**CH$_3$**COO-6 indole	2.26(s)	20.9
NH**CH$_2$**CH$_2$CH$_2$CH$_2$CH$_2$CH$_2$NH	3.32(m)	39.3
**CH-3** indole	7.12(bs)	102.8
**CH-4** indole	7.45(bs)	106.5
**CH-7** indole	7.22(bs)	115.2
CH$_2$**NH**CO	8.51(t,6)	
**NH** indole	11.72(bs)	
**C9** indole		124.9
**C2** indole		133.9
**C8** indole		134.0
**C6** indole		136.8
**C5** indole		139.3
NH**C**O		161.0
CH$_3$**C**OO-5 indole		169.2
CH$_3$**C**OO-6 indole		169.4

*Resonance assignment based on 2D NMR spectra (not shown)

.

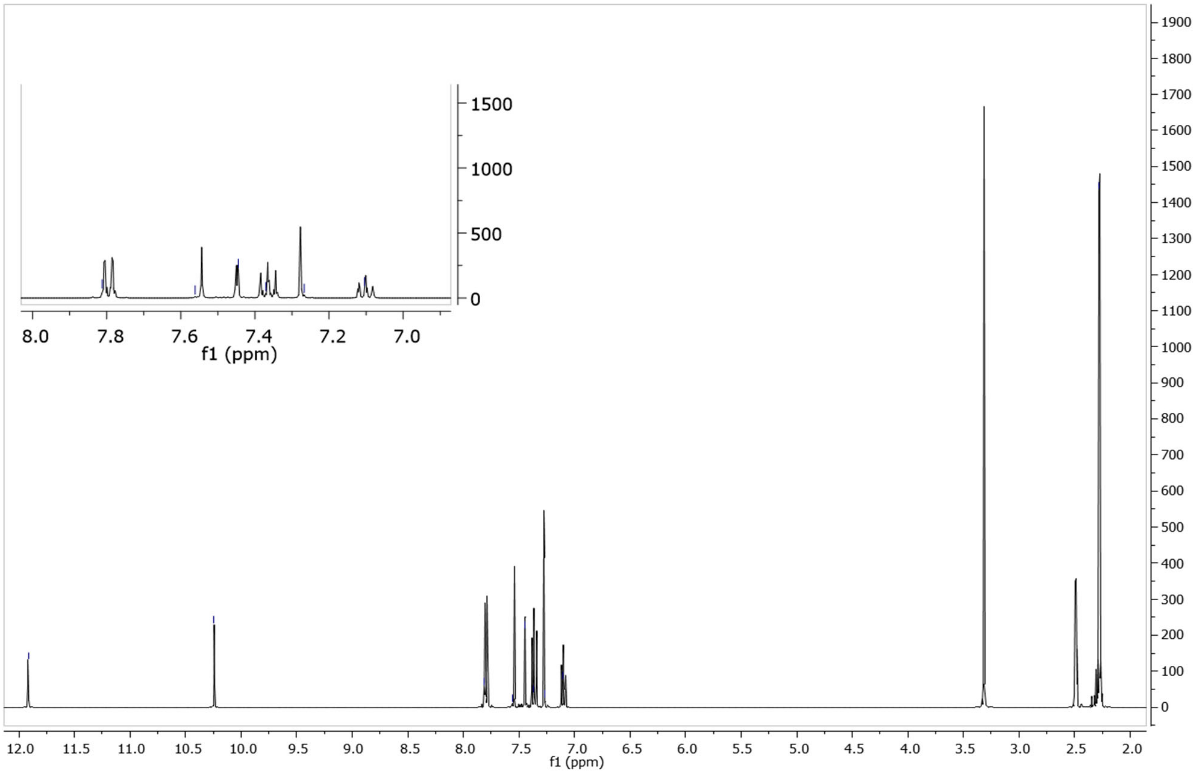

Figure S10. ^{1}H NMR spectrum of 5,6-diacetoxy-*N*-phenyl-1*H*-indole-2-carboxamide of DHICA (**4**) (DMSO-d_6).

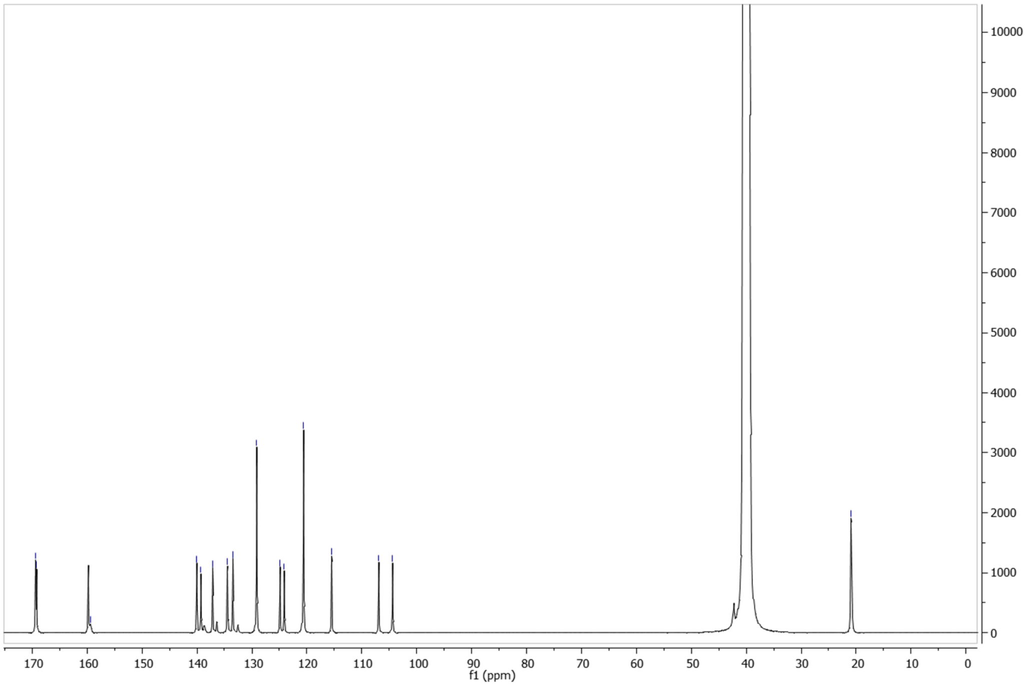

Figure S11. ^{13}C NMR spectrum of 5,6-diacetoxy-*N*-phenyl-1*H*-indole-2-carboxamide of DHICA (**4**) (DMSO-d_6).

Table S4. NMR spectral data (ppm) for **4.**

	**[1]H (J, Hz)**	**[13]C**
**CH_3**COO-5 indole	2.27(s)	20.8
**CH_3**COO-6 indole	2.28(s)	20.9
**CH**-aniline	7.10(t,8.0)	124.1
**CH-7** indole	7.27(t,0.8)	106.8
**CH**-aniline	7.37(t,8.0)	120.6
**CH-3** indole	7.44(dd, 2.4,0.8)	104.4
**CH-4** indole	7.55(bs)	115.4
**CH**-aniline	7.88(d,2.0)	129.2
aniline **NH**CO	10.24(s)	
**NH** indole	11.92(bs)	
**C9** indole		124.5
**C2** indole		133.4
**C8** indole		134.5
**C6** indole		137.1
**C5** indole		139.3
**C** aniline		140.0
NH**C**O		159.9
CH_3**C**OO-5 indole		169.2
CH_3**C**OO-6 indole		169.4

*Resonance assignment based on 2D NMR spectra (not shown)

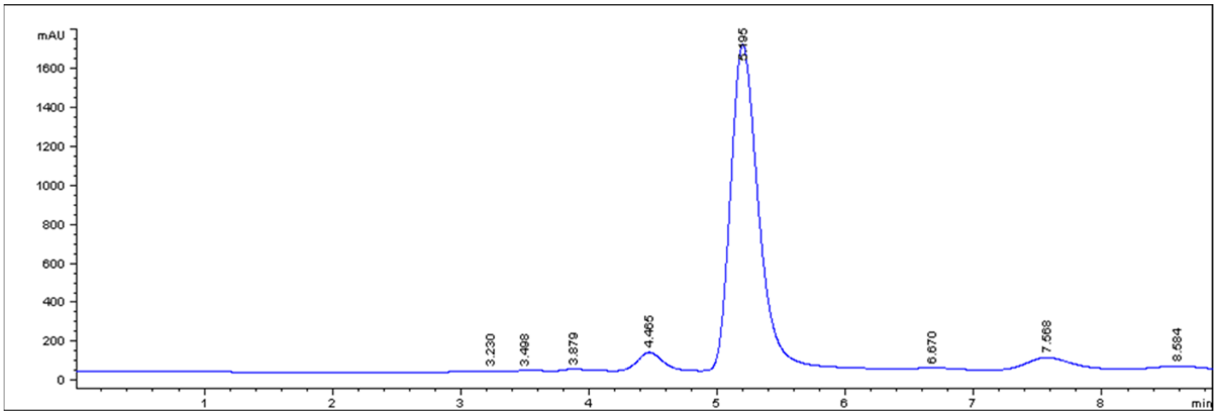

Figure S12. Chromatogram of deacetylated **1** (λ = 300 nm).

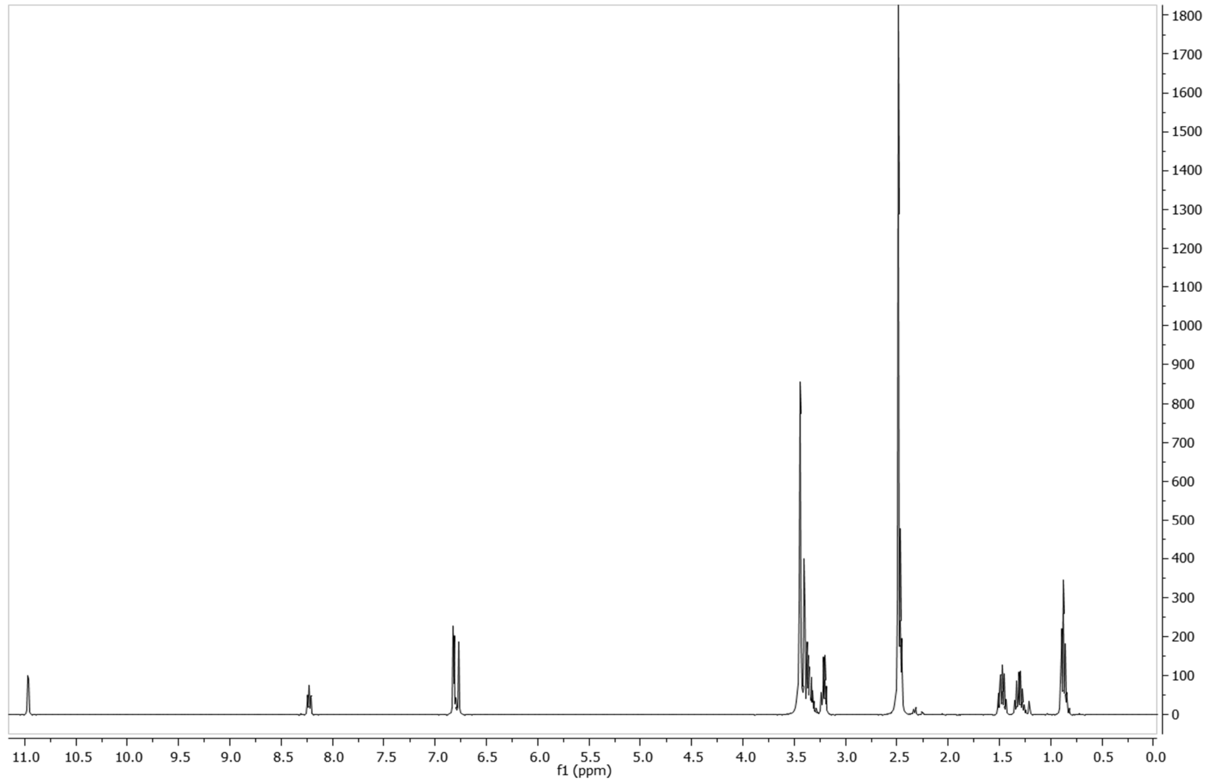

Figure S13. ^{1}H NMR spectrum of deacetylated **1** (DMSO-d_6).

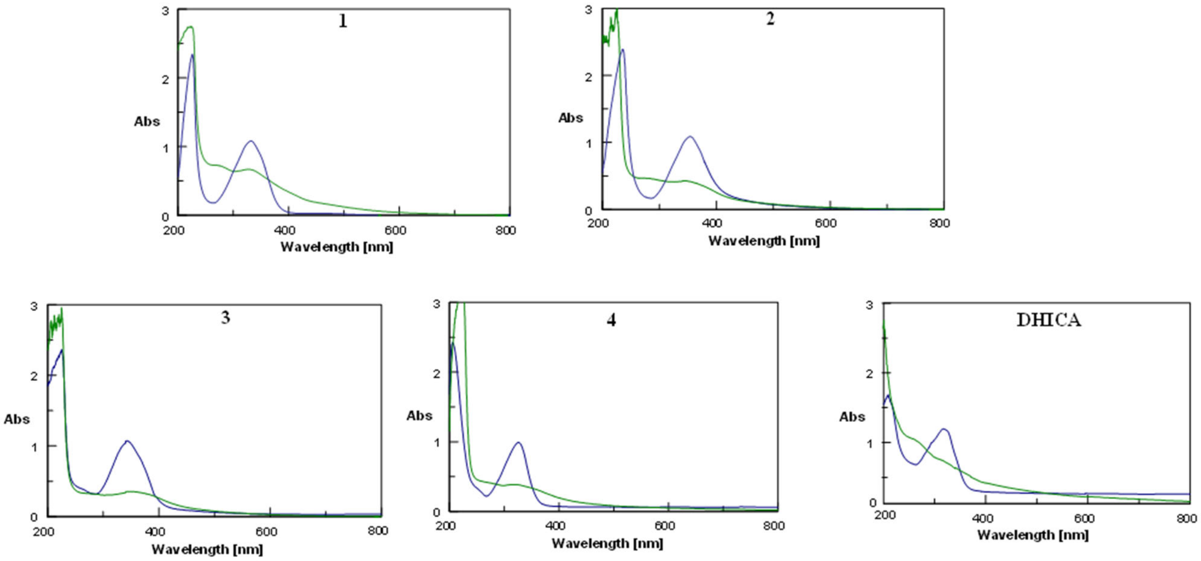

Figure S14. UV-Vis spectrophotometric monitoring of the course of the aerial oxidation of DHICA carboxyamides at t 0 h (blu), t24 h (green).

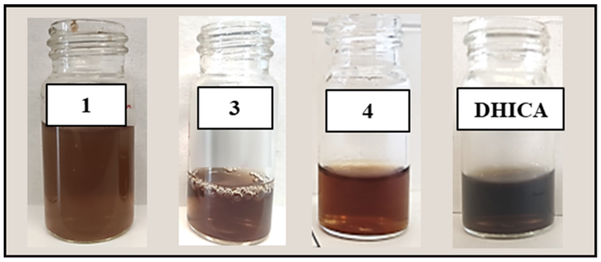

Figure S15. Digital photos of pigments as obtained from DHICA carboxyamides in carbonate buffer at pH 9 after 24 h under stirring.

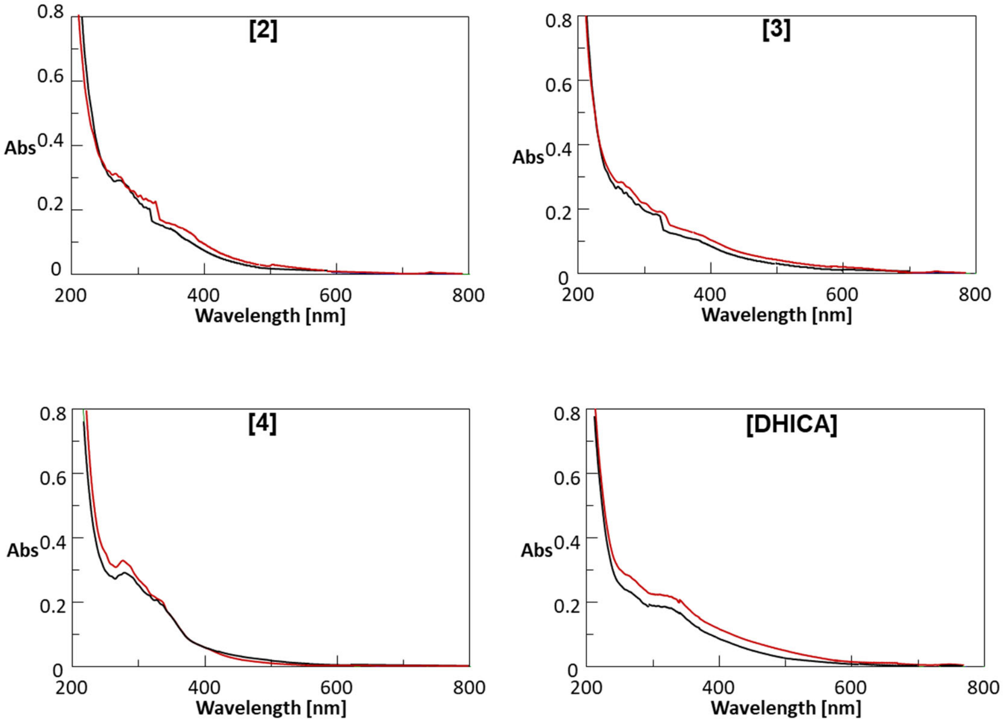

Figure S16. UV-vis spectrum prior and after filtration of the ethanol solution (1mg/mL) of carboxamides and DHICA.
